# Pathogenicity of 2 Porcine Deltacoronavirus Strains in Gnotobiotic Pigs

**DOI:** 10.3201/eid2104.141859

**Published:** 2015-04

**Authors:** Kwonil Jung, Hui Hu, Bryan Eyerly, Zhongyan Lu, Juliet Chepngeno, Linda J. Saif

**Affiliations:** Ohio State University, Wooster, Ohio, USA

**Keywords:** porcine deltacoronavirus, PDCoV, coronavirus, viruses, strains, strain OH-FD22, strain OH-FD100, pathogenicity, pigs, gnotobiotic pigs, PCR

## Abstract

To verify whether porcine deltacoronavirus infection induces disease, we inoculated gnotobiotic pigs with 2 virus strains (OH-FD22 and OH-FD100) identified by 2 specific reverse transcription PCRs. At 21–120 h postinoculation, pigs exhibited severe diarrhea, vomiting, fecal shedding of virus, and severe atrophic enteritis. These findings confirm that these 2 strains are enteropathogenic in pigs.

Porcine epidemic diarrhea virus (PEDV) (family *Coronaviridae,* genus *Alphacoronavirus*) was discovered in the United States in May 2013. The virus has now spread nationwide and caused a high number of deaths among suckling pigs (*1*,*2*). In regions of the United States to which PEDV is epidemic, a new coronavirus, genetically distinct from PEDV, porcine deltacoronavirus (PDCoV) (genus *Deltacoronavirus*), has been simultaneously and frequently detected in diarrheic fecal samples from pigs (*3*–*5*).

The clinical role and disease severity of PDCoV in the field is reportedly less than that of PEDV (*6*). To confirm the role of PDCoV as an enteric viral pathogen and understand disease progression, we studied the pathogenicity of 2 strains of PDCoV (OH-FD22 and OH-FD100) in gnotobiotic pigs. We developed in situ hybridization and immunofluorescence staining methods to verify the tissue sites of PDCoV replication in infected pigs.

## The Study

In February and July 2014, intestinal contents were obtained from young nursing piglets with diarrhea on farms in Ohio, USA. PDCoV strains OH-FD22 and OH-FD100 were detected in samples by using a TaqMan quantitative reverse transcription RT-PCR (qRT-PCR) specific for the membrane gene (nt 23395–23466) as reported (*7*), or an RT-PCR specific for the PDCoV membrane gene (nt 23111–23651) based on the sequence of PDCoV strain (USA/Illinois121/2014; GenBank accession no. KJ481931), as described in the in situ hybridization method. The partial membrane gene sequences of OH-FD22 and OH-FD100 were identical to that of USA/Illinois121/2014. Samples were negative for PEDV, rotavirus groups A–C, transmissible gastroenteritis virus (TGEV)/porcine respiratory coronavirus (PRCV), and caliciviruses (noroviruses, sapoviruses, and St. Valerien–like viruses) by RT-PCR as reported (*8*). The samples were bacteriologically sterilized by using 0.22-μm syringe filters and then prepared as inoculum.

Near-term gnotobiotic pigs were delivered aseptically by hysterectomy from 2 specific pathogen–free sows (*9*). Seven 11- to 14-day-old pigs were randomly assigned to a PDCoV-inoculated group (pigs 1–5) or as negative controls (pigs 6 and 7). Pigs 1–3 and pigs 4 and 5 were inoculated orally with 8.8 log_10_ genomic equivalents (GEs) of PDCoV strain OH-FD22 and 11.0 log_10_ GEs of OH-FD100, respectively. Clinical signs were monitored hourly. Pig 2 was monitored for long-term clinical signs and virus shedding until day postinoculation (dpi) 23. Pigs were euthanized for pathologic examination at 24–48 h or >48 h after onset of clinical signs. All animal-related experimental protocols were approved by the Ohio State University Institutional Animal Care and Use Committee.

Fecal or rectal swab samples were prepared as described (*8*,*9*). Virus RNA was extracted by using the Mag-MAX Viral RNA Isolation Kit (Applied Biosystems, Foster City, CA, USA) according to the manufacturer’s instructions. Titers of virus shed in feces were determined by using the qRT-PCR and the OneStep RT-PCR Kit (QIAGEN, Valencia, CA, USA) as reported (*7*). A standard curve was generated by using the PCR amplicon (nt 23111–23651) of strain OH-FD22. The detection limit of the qRT-PCR was 10 GEs/reaction, which corresponded to 4.6 log_10_ and 3.6 log_10_ GEs/mL of PDCoV in fecal and serum samples, respectively.

Small and large intestinal tissues and other major organs (lung, liver, heart, kidney, spleen, and mesenteric lymph nodes) were examined. Mean jejunal ratios of villous height to crypt depth were measured as reported (*8*). For PDCoV RNA detection in formalin-fixed, paraffin-embedded tissues, a nonradioactive digoxigenin-labeled cDNA probe specific for the 541-bp virus membrane gene sequence (nt 23111–23651), amplified with the primers forward 5′-CGCGTAATCGTGTGATCTATGT-3′ and reverse 5′-CCGGCCTTTGAAGTGGTTAT-3′, was used for in situ hybridization as described (*10*). Reverse transcription was conducted at 50°C for 30 min, followed by denaturation at 94°C for 5 min; 35 cycles at 94°C for 40 s, 55°C for 40 s, and 72°C for 1 min; and final extension at 72°C for 7 min.

OH-FD22–infected pig 2 was immunized intramuscularly with OH-FD22 from the gnotobiotic pig–passaged intestinal contents that were semipurifed by sucrose gradient ultracentrifugation (*11*) and mixed with complete and incomplete Freund’s adjuvants at dpi 30 and dpi 44 (*11*). Immunofluorescence staining was performed on frozen or formalin-fixed, paraffin-embedded tissues as described (*8*,*9*) by using hyperimmune gnotobiotic pig antiserum against OH-FD22. Tissues from control pigs 6 and 7 and PEDV-infected gnotobiotic pigs (*8*) were used negative controls for in situ hybridization/immunofluorescence staining.

**Figure 1:**
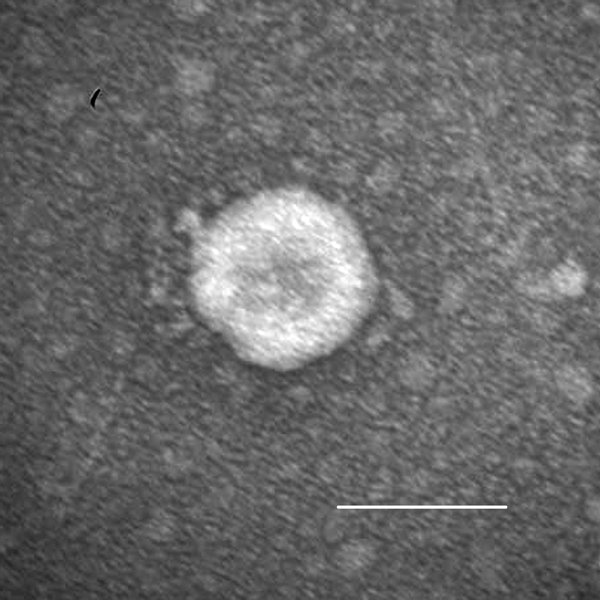
Porcine deltacoronavirus (OH-FD22) particle detected in intestinal contents from a gnotobiotic pig. The sample was negatively stained with 3% phosphotungstic acid. Scale bar indicates 100 nm.

Acute, severe, watery diarrhea, vomiting, or both developed in all inoculated pigs. Clinical signs developed at hour postinoculation (hpi) 21–24, regardless of the inoculum strain or dose (Table 1). At hpi 96–120, pig 1 exhibited severe dehydration, loss of bodyweight, and lethargy. Pig 2, which was followed up longer, showed diarrhea until dpi 7. All inoculated pigs exhibited onset of clinical disease similar to that of infection with PEDV strain PC21A (6.3–9.0 log_10_ GEs/pig) in gnotobiotic pigs (*8*). Immune electron microscopy with hyperimmune serum to PDCoV from a gnotobiotic pig showed only PDCoV particles in the intestinal contents (Figure 1). For pig-passaged OH-FD22 and OH-FD100 samples, RT-PCR results were negative for PEDV, rotavirus groups A–C, TGEV/PRCV, and caliciviruses. Detection of fecal virus shedding at hpi 24 coincided with onset of clinical signs (Table 1) in pigs 2–5. In pig 1, which showed only vomiting at hpi 24, fecal shedding occurred at hpi 48 at the onset of diarrhea.

**Table 1:** Fecal shedding of virus and clinical signs after inoculation of gnotobiotic pigs with PDCoV strains OH-FD22 and OH-FD100*

Pig status, no. (age at inoculation, d)	Virus strain	Oral inoculum, log_10_ GEs	Fecal shedding, log_10_ GE/mL by hpi†						Clinical signs (onset hpi)
0	24	48	72	96	120
PDCoV-inoculated									
1 (14)	OH-FD22	8.8	<4.6	<4.6	8.1	7.3	7.4	6.7‡	Diarrhea/vomiting (21–24)§
2 (14)	OH-FD22	8.8	<4.6	6.2	8.5	8.4	6.1	7.6	Diarrhea/vomiting (21–24)
3 (14)	OH-FD22	8.8	<4.6	8.4	8.2	8.8‡	–	–	Diarrhea/vomiting (21–24)
4 (11)	OH-FD100	11.0	<4.6	7.1	6.0	ND	ND‡	–	Diarrhea/vomiting (22–24)
5 (11)	OH-FD100	11.0	<4.6	8.2	8.5	ND‡	–	–	Diarrhea/vomiting (22–24)
Negative control									
6 (16)¶	None	None	<4.6	<4.6	<4.6	<4.6‡	–	–	None
7 (17)¶	None	None	<4.6	<4.6	<4.6	<4.6‡	–	–	None

*PDCoV, porcine deltacoronavirus; GE, genome equivalent; hpi, hour postinoculation; –, no result (pig euthanized); ND, not determined. †Detected by real-time quantitative reverse transcription PCR. The detection limit of the PCR was <4.6 log_10_ GE/mL for a fecal sample and <3.6 log_10_ GE/mL for a serum sample. ‡Pig was euthanized. §Pig 1 vomited at days postinoculation 21–24 and then had diarrhea. ¶At euthanasia.

**Figure 2:**
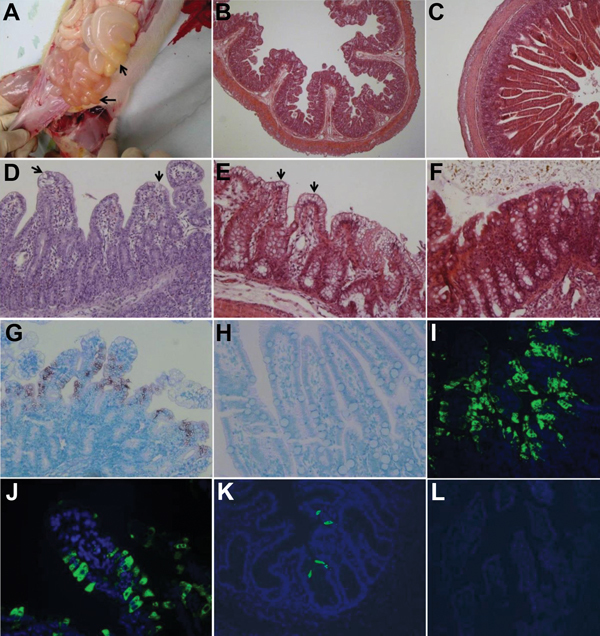
Intestinal changes in gnotobiotic pigs inoculated with porcine deltacoronavirus (PDCoV) strains OH-FD22 (panels A, B, E, G, I, and J) and OH-FD100 (panels D and K). A) Intestine of pig 3 at hour postinoculation (hpi) 72 (48–51 h after onset of clinical signs), showing thin and transparent intestinal walls (duodenum to colon) and accumulation of large amounts of yellow fluid in the intestinal lumen (arrows). B) Jejunum of pig 3 at hpi 72 (48–51 h after onset of clinical signs), showing acute diffuse, severe atrophic jejunitis (original magnification ×40). C) Jejunum of noninoculated pig 7, showing normal villous epithelium (original magnification ×80). D) Jejunum of pig 4 at hpi 96 (72–74 h after onset of clinical signs), showing acute diffuse, severe atrophic jejunitis with mild cytoplasmic vacuolation at the tips of villi (arrows) (original magnification ×200). E) Colon of pig 3 at hpi 72 (48–51 h after onset of clinical signs), showing mild cytoplasmic vacuolation of superficial epithelial cells (arrows) (original magnification ×200). F) Colon of noninoculated pig 7, showing normal colonic epithelium (original magnification ×200). G) Jejunum of pig 3 at hpi 72 (48–51 h after onset of clinical signs), showing that epithelial cells lining the atrophied villi are positive for PDCoV RNA (original magnification ×200). H) Jejunum of noninoculated pig 6, showing absence of PDCoV RNA-positive cells and background staining (original magnification ×200). I) Jejunum of pig 3 at hpi 72 (48–51 h after onset of clinical signs), showing large numbers of PDCoV antigen–positive cells in the epithelium of atrophied villi (original magnification ×200). J) Jejunum of pig 3 at hpi 72 (48–51 h after onset of clinical signs), showing localization of PDCoV antigens in cytoplasm of columnar epithelial cells (original magnification ×400). K) Cecum of pig 4 at hpi 96 (72–74 h after onset of clinical signs), showing a few PDCoV antigen–positive cells in the epithelium (original magnification ×200. L) Jejunum of noninoculated pig 6, showing absence of immunofluorescence-stained cells and background staining (original magnification ×200). Nuclei were stained with blue-fluorescent 4′, 6-diamidino-2-phenylindole dihydrochloride. Hematoxylin and eosin staining in panels B–F; in situ hybridization staining in panels G and H; immunofluorescence staining in panels I–L.

Macroscopic examination showed that all infected pigs had PEDV-like lesions characterized by thin and transparent intestinal walls (proximal jejunum to colon) and accumulation of large amounts of yellow fluid in the intestinal lumen (Figure 2, panel A). The stomach was filled with curdled milk. Other internal organs appeared normal. Histologic lesions included acute diffuse, severe atrophic enteritis (Figure 2, panels B, D) and mild vacuolation of superficial epithelial cells in cecum and colon (Figure 2, panel E). The mean jejunal ratios of villous height to crypt depth of infected pigs 3–5 at hpi 72–120 ranged from 1.4 to 3.6 (Table 2), which were similar to those in gnotobiotic pigs experimentally infected with PEDV strain PC21A (*8*). Clinical signs or lesions did not develop in negative control pigs during the experiment (Figure 2, panels C, F).

**Table 2:** Histopathologic findings after inoculation of gnotobiotic pigs with PDCoV strains OH-FD22 and OH-FD100*

Pig status, no.	hpi at euthanasia	Mean VH:CD	RNA detection in formalin-fixed, paraffin-embedded tissues/antigen detection in frozen tissues (ISH/IF results)†			
			Duodenum	Jejunum	Ileum	Cecum/colon
PDCoV-inoculated						
1	120	ND	−/−	+/+++	+/+++	–/±
2‡	NA	NA	NA	NA	NA	NA
3	72	3.6 (1.7)	–/±	++/+++	++/+++	±/±
4	96	1.4 (0.1)	−/−	++/+++	++/+++	–/±
5	72	1.6 (0.5)	–/+	++/+++	++/+++	–/±
Negative control						
6	NA	5.8 (0.9)	−/−	−/−	−/−	−/−
7	NA	5.6 (0.5)	−/−	−/−	−/−	−/−

*PDCoV, porcine deltacoronavirus; hpi, hour postinoculation; VH:CD, ratio of villous height to crypt depth; ISH, in situ hybridization; IF, immunofluorescence staining; ND, not determined because of autolysis at tips of jejunal villi; NA, not applicable. †Detected by ISH or IF staining. –, no cells showed staining; +, 1%–29% of epithelial cells showed staining; +++, 60%–100% of epithelial cells showed staining as described (*12*); ±, <1% of epithelial cells showed staining; ++, 30%–59% of epithelial cells showed staining. Other internal organs showed no ISH or IF staining. ‡Pig 2 was used for production of hyperimmune gnotobiotic antiserum against PDCoV strain OH-FD22.

In situ hybridization–positive or immunofluorescence-stained cells were observed mainly in the villous epithelium of small (duodenum to ileum) and large intestines (Table 2; Figure 2, panels G, I–K). Immunofluorescence was confined to the cytoplasm of villous epithelial cells (Figure 2, panel J) and was infrequently observed in crypt epithelial cells. No other internal organs of infected pigs showed in situ hybridization–positive or immunofluorescence-positive staining. In situ hybridization–positive or immunofluorescence-stained cells were not detected in negative control pigs (Figure 2, panels H, L) and PEDV-infected gnotobiotic pigs.

Under the experimental conditions used, no PDCoV-inoculated pigs at hpi 72–168 had detectable virus RNA (<3.6 log_10_ GEs/mL) in serum. However, viremia was detected frequently in symptomatic PEDV-infected pigs (*8*,*13*).

## Conclusions

Since 2013–2014, newly emerged PEDV and PDCoV have spread throughout the United States and caused a high number of pig deaths (*1*,*2*,*6*), but no studies of the pathogenicity of PDCoV have been reported. Our data confirm that PDCoV strains OH-FD22 and OH-FD100 are enteropathogenic and acutely infect the entire intestine. However, the jejunum and ileum are the primary sites of infection. PDCoV infection caused severe atrophic enteritis accompanied by severe diarrhea, vomiting, or both. These clinical and pathologic features in PDCoV-infected pigs resemble those of PEDV and TGEV infections. Differential diagnosis of PDCoV, PEDV, and TGEV is critical to control virus epidemic diarrhea on swine farms in the United States.
